# Chronic inflammation in benign prostatic hyperplasia: Pathophysiology and treatment options

**DOI:** 10.1111/iju.15518

**Published:** 2024-06-27

**Authors:** So Inamura, Naoki Terada

**Affiliations:** ^1^ Department of Urology, Faculty of Medical Sciences University of Fukui Eiheiji Japan

**Keywords:** benign prostatic hyperplasia, chronic inflammation, microbiome, pelvic ischemia, treatments, urine reflux

## Abstract

Benign prostatic hyperplasia, a prevalent condition in aging men, is characterized by the proliferation of prostatic epithelial and stromal cells, which leads to bladder outlet obstruction and the exacerbation of lower urinary tract symptoms. There is increasing evidence that chronic prostatic inflammation contributes to the pathogenesis and progression of benign prostatic hyperplasia. This review explores the complex relationship between chronic inflammation and benign prostatic hyperplasia, focusing on the underlying mechanisms, clinical implications, and current therapeutic approaches. The pathophysiology of benign prostatic hyperplasia is multifaceted, involving factors such as hormonal changes, hypoxia, urine reflux into prostatic ducts and stroma, autoimmune responses, and infection‐induced inflammation. Inflammatory cytokines, particularly interleukin‐17 and interleukin‐8, may play key roles in tissue remodeling and smooth muscle contraction within the prostate, thereby influencing benign prostatic hyperplasia progression. Current therapies for benign prostatic hyperplasia include α1‐blockers, phosphodiesterase 5 inhibitors, 5α‐reductase inhibitors, and plant‐based treatments (e.g., pollen extract). These therapies aim to alleviate symptoms by reducing prostatic inflammation, improving blood flow, and inhibiting hormonal pathways involved in prostatic enlargement. However, patients with chronic prostatic inflammation often experience more severe lower urinary tract symptoms and may be resistant to conventional treatments. This resistance has prompted the exploration of alternative therapies targeting inflammation. Chronic prostatic inflammation plays a central role in the pathogenesis and severity of benign prostatic hyperplasia. An understanding of its mechanisms will enable the development of more effective treatments to improve the quality of life among patients with benign prostatic hyperplasia.

**Abbreviations & Acronyms**
5ARI: 5α‐reductase inhibitor
BOOI: bladder outlet obstruction index
BPH: benign prostatic hyperplasia
CP/CPPS: chronic prostatitis with chronic pelvic pain syndrome
DM: diabetes mellitus
HEV: high endothelial venule
IPSS: International Prostate Symptom Score
LUTDs: lower urinary tract diseases
LUTS: lower urinary tract symptoms
NIH‐CPSI: National Institute of Health Chronic Prostatitis Symptom Index
NSAIDs: Nonsteroidal anti‐inflammatory drugs
PAP: prostatic acid phosphatase
PSA: prostate‐specific antigen
RGS4: regulator of G protein signaling 4

## INTRODUCTION

Chronic inflammation, a reaction to sustained harmful stimulus exposure characterized by lymphocyte infiltration into inflamed sites and eventual tissue destruction,^1^ plays key roles in various diseases, including cancer.^2^

Recently, it has become clear that chronic inflammation is integral to the pathogenesis of lower urinary tract diseases (LUTDs). In particular, chronic inflammation of the prostate has been identified as an exacerbating factor in benign prostatic hyperplasia (BPH)^3^ and chronic prostatitis with chronic pelvic pain syndrome (CP/CPPS).^4^ However, the mechanisms linking chronic inflammation and pathogenesis in these diseases are unclear. Additionally, despite the importance of chronic inflammation in lower urinary tract symptoms (LUTS), there is no gold‐standard therapy for chronic inflammation in the lower urinary tract.

BPH and CP are often regarded as overlapping diagnoses. Nickel et al. evaluated 3700 patients with BPH and LUTS, among whom 688 (18.6%) reported that pelvic pain/discomfort on ejaculation was their main concern; patients with painful ejaculation also had more severe LUTS.^5^ Thus, a mechanistic understanding of chronic prostatic inflammation might help researchers achieve crucial insights and identify new treatments for prostatic diseases.

In this review, we discuss the pathogenesis of chronic prostatic inflammation and its impact on LUTS based on the results of our studies and other current research.

## ASSOCIATION BETWEEN CHRONIC INFLAMMATION AND BPH

The specific mechanisms underlying the links between chronic prostatic inflammation and LUTDs, as well as the precise mechanisms of BPH progression, are not fully understood. Factors associated with BPH symptoms include prostatic enlargement and prostatic smooth muscle tension.

## RELATIONSHIP BETWEEN PROSTATIC ENLARGEMENT AND PROSTATIC INFLAMMATION

Several factors contribute to prostatic enlargement, including hormonal changes^6^ and chronic inflammation.^7^ Clinical and basic research studies have explored the effects of prostatic inflammation on prostatic enlargement. The REDUCE (REduction by DUtasteride of prostate Cancer Events) trial is a large‐scale clinical trial that examined whether dutasteride (a dual 5α‐reductase [5ARI] inhibitor) could reduce the risk of biopsy‐detectible prostate cancer among men with prostate cancer risk (*n* = 8224).^8^ Nickel et al. reported that prostate volume was significantly greater in patients with chronic prostatic inflammation than in patients without prostatic inflammation (46.5 vs. 43.4 mL, *p* < 0.0001) in the REDUCE trial.^7^ Li et al. also demonstrated that patients with histological prostatitis had greater prostate volume (patients with vs. without inflammation: 61.19 vs. 52.28 mL), more severe LUTS (23.94 vs. 21.86), and a higher risk of acute urinary retention (66.2% vs. 33.3%), suggesting that histological prostatitis is closely related to BPH onset and clinical progression.^9^ In contrast, our previous study showed no correlation between high endothelial venule (HEV)‐like vessel number and prostate volume (correlation coefficient: −0.1128, *p* = 0.152), whereas the HEV‐like vessel number was correlated with the bladder outlet obstruction index (BOOI) (correlation coefficient: 0.2699, *p* = 0.0105). HEV‐like vessels, identified by MECA‐79 immunostaining, are essential for lymphocyte recruitment and infiltration from the systemic circulation into inflamed sites; the HEV‐like vessel number reportedly reflects the degree of chronic inflammation.^10^, ^11^ The above discrepancies may be due to differences in prostatic inflammation assessment and cohort characteristics. The studies of Nickel et al. and Li et al. evaluated prostatic inflammation using hematoxylin and eosin–stained tissue, whereas our study focused on HEV‐like vessels. In terms of cohort characteristics, the REDUCE trial primarily included patients with relatively mild BPH, whereas the latter two cohorts were composed of surgical cases (i.e., presumably patients with severe BPH).

Thus, in some clinical studies, the results indicate that prostatic inflammation increases prostate volume; the findings in other studies suggest that such inflammation is irrelevant. Even when the results indicate that inflammation increases prostate volume, there is considerable variation among studies, but differences in prostate volume between groups are small, and the clinical implications of links between inflammation and prostate volume should be carefully examined.

Conversely, some basic research has demonstrated that prostatic inflammation contributes to prostatic enlargement. Cytokines and chemokines such as interleukin (IL)‐17, interferon‐γ, and IL‐8 are involved in the proliferation of prostatic tissue. Several studies have demonstrated elevated expression levels of IL‐17 in T cells, interferon‐γ in basal and stromal cells, and IL‐8 in epithelial cells.^12^ IL‐8 reportedly can induce fibroblast growth factor (FGF)‐2 expression in the prostate; FGF‐2 promotes the abnormal proliferation of prostatic tissue.^13^ These findings indicated that prostatic inflammation may be involved in a cycle of tissue injury and remodeling within the prostate, driving the increase in prostate volume. Kobayashi et al. evaluated the relationship between changes in prostate volume and levels of inflammatory cytokines in aging rats with and without diabetes mellitus (DM) (OLETF and LETO rats, respectively). In both types of rats, the prostate volume and levels of inflammatory cytokines increased with age; treatment with tadalafil led to decreases in both.^14^

Although basic research results suggest that inflammatory cytokines induce prostate growth factor production leading to increased prostate volume, clinical investigations indicate that inflammation‐induced volume increases may be limited. Direct comparisons between basic and clinical studies require caution due to differences in prostatic inflammation assessment methods. Thus, further investigations are necessary.

## PROSTATIC INFLAMMATION AND BLADDER OUTLET OBSTRUCTION (BOO)

The relationship between prostate volume and prostatic inflammation remains a topic of debate; however, there have been some reports supporting a link between prostatic inflammation and BOO. In the REDUCE trial, the risk of acute urinary retention was higher among patients with chronic inflammation than among patients without such inflammation (hazard ratios: 1.6–1.8, *p* = 0.001).^15^ Moreover, Li et al. observed a higher number of acute urinary retention cases among patients with inflammation than among patients without such inflammation (66.2% vs. 33.3%).^9^ Additionally, our study (described above) showed a correlation between the severity of chronic prostatic inflammation and BOOI. Thus, prostatic inflammation may be involved in functional obstruction due to prostatic smooth muscle cell contraction rather than mechanical obstruction.

Various inflammatory cytokines promote smooth muscle tension in other organs. Akiho et al. reported that IL‐17A induced hypercontractility within intestinal smooth muscle cells through a signaling cascade involving IκBζ and regulator of G protein signaling 4 (RGS4).^16^ Govindaraju et al. demonstrated that IL‐8 strongly induced the contraction of airway smooth muscle cells in patients with cystic fibrosis.^17^ Prostatic smooth muscle cells might respond to inflammatory cytokines in a similar manner. However, to our knowledge, no studies have demonstrated the effects of cytokines on prostatic smooth muscle cell contraction. Clarification of the relationship between prostatic smooth muscle cells and inflammatory cytokines could help to develop new therapeutic approaches for BPH.

## RELATIONSHIP BETWEEN STORAGE DYSFUNCTION AND PROSTATIC INFLAMMATION

In addition to voiding symptoms, some studies (including our own) showed that chronic prostatic inflammation was associated with storage symptoms. The mechanisms underlying the relationship between storage dysfunction and BPH are complex and not fully understood. Yokoyama et al. demonstrated that urethral stimulation with prostaglandin enhances the micturition reflex^18^; this result suggests that urethral sensory nerves have an important role in the onset of storage symptoms.

Recent research has revealed cross‐sensitization between the prostate and bladder. Funahashi et al. demonstrated prostate‐to‐bladder cross‐sensitization and the impact of prostatic inflammation on storage symptom exacerbation using rat models of nonbacterial prostatic inflammation.^19^ They labeled bladder afferent neurons by injecting Fast Blue into the bladder wall and prostate afferent neurons by injecting 0.2% 1,1′‐dioctadecyl‐3,3,3′,3′‐tetramethylindocarbocyanine perchlorate (DiI) into the prostate, then analyzed the distributions of those neurons. Some neurons in the dorsal root ganglia exhibited both Fast Blue and DiI labeling, indicating that some nerves in the prostate and bladder branch from the same upstream nerve fibers. Additionally, Funahashi et al. evaluated bladder function in rats with and without prostatic inflammation.^19^ Using cystometry, they demonstrated that the bladder contraction interval was shorter in rats with prostatic inflammation than in rats without prostatic inflammation; they also found that prostatic inflammation led to increased nerve growth factor expression, altered ion channel expression, and enhanced bladder afferent nerve excitability. Taken together, these results suggest that inflammatory stimuli in the prostate or urethra induce bladder overactivity through various mechanisms.

**FIGURE 1:**
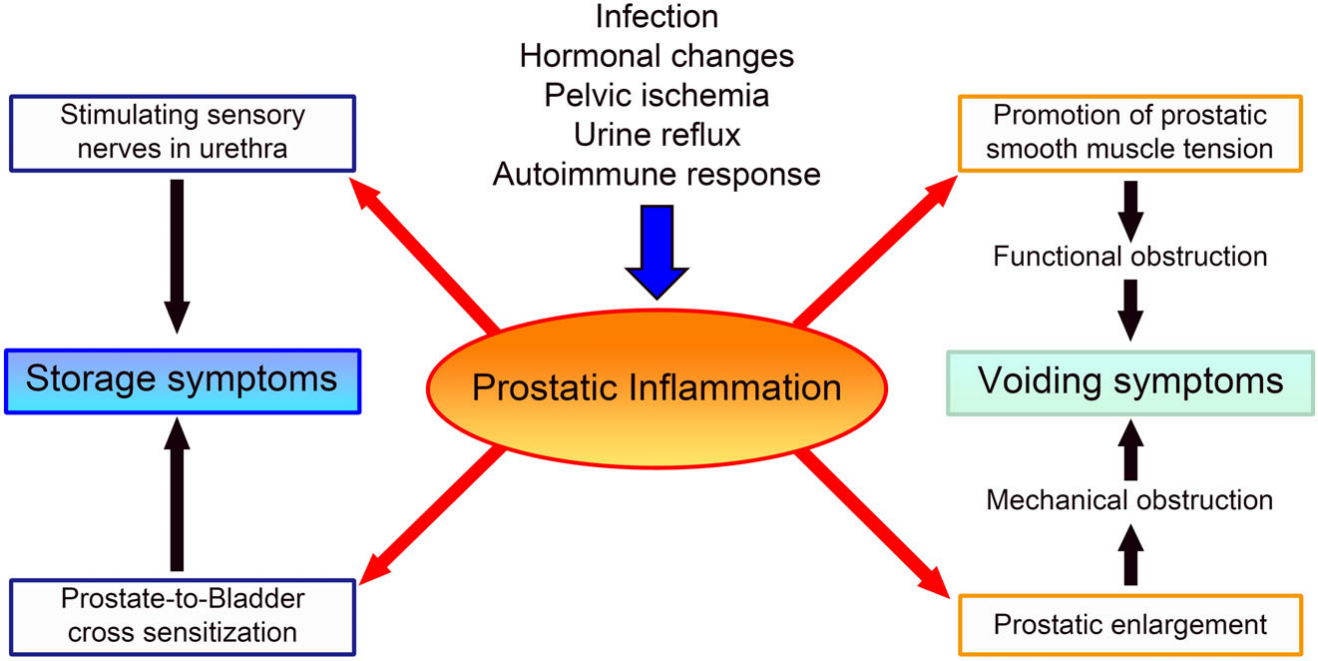
The relationship between prostatic inflammation and LUTS. Prostatic inflammation influences voiding and storage symptoms through various mechanisms. Prostatic enlargement and increased prostatic smooth muscle tension are considered the main mechanisms that worsen voiding symptoms in patients with prostatic inflammation. Additionally, prosthetic inflammation may affect the neurological control of storage function and worsen storage symptoms in patients with prostatic inflammation.

As summarized above, prostatic inflammation affects BPH severity in terms of prostatic enlargement, BOO exacerbation, and storage dysfunction (Figure 1). As a result, LUTS exacerbation and resistance to drug therapy often occur in patients with BPH with prostatic inflammation.

## PATHOPHYSIOLOGY OF PROSTATIC INFLAMMATION

Prostatic inflammation arises from multiple factors, including infection, autoimmune responses, hormonal changes, pelvic ischemia, and urine reflux into prostatic ducts. Prostatic inflammation is likely caused by a combination of overlapping factors, rather than a single factor.

## INFECTION

Viruses involved in the pathophysiology of prostatic inflammation include human papilloma virus, herpes simplex virus 2, cytomegalovirus, and human herpesvirus 8. Bacteria presumably contributing to prostatic inflammation include gram‐negative bacilli and sexually transmitted infections caused by Neisseria gonorrhoeae, Chlamydia trachomatis, Treponema pallidum, and Trichomonas vaginalis.

Recent research has highlighted the complex relationships between prostatic inflammation and the gut and urinary microbiomes in various urological diseases, including benign and malignant prostate conditions. Bajic et al. examined the relationship between BPH and human microbiomes; their 16S rRNA sequencing and expanded quantitative urine culture analyses identified unique microbiomes in the distal (anterior urethral) and proximal (bladder) regions of the male lower urinary tract. Significant qualitative and quantitative differences were evident, particularly between highly symptomatic patients with BPH undergoing surgical treatment and such patients with minimal or no symptoms.^20^ Takezawa et al. used 16S rRNA metagenomics to analyze the gut microbiomes of 128 patients who underwent prostate biopsies and were not diagnosed with prostate cancer. The results showed that the ratio of Firmicutes to Bacteroidetes was significantly higher in patients with prostatic enlargement than in patients without prostatic enlargement.^21^ These findings suggest a connection between the urinary microbiome and male LUTS.

## HORMONAL INFLUENCE

Generally, androgens are considered key factors in prostatic enlargement. Testosterone is converted to dihydrotestosterone and acts on androgen receptors to promote cell division within the prostate, contributing to increased prostate volume.^6^ The anti‐inflammatory effects of androgens have been identified in various organs. For example, dihydrotestosterone can suppress the production of cytokines and chemokines such as IL‐1, IL‐6, and IL‐8 by modulating NF–κB or IκB signaling.^22^ However, few studies have evaluated the effects of androgen on prostatic inflammation. Thus, we suspect that altered androgen levels in the prostate affect the severity of prostatic inflammation; further studies are necessary to explore this hypothesis.

## PELVIC ISCHEMIA

Pelvic ischemia may play a pivotal role in chronic prostatic inflammation. Local hypoxia causes inflammation, inducing cytokine and growth factor secretion. The onset of hypoxia contributes to reactive oxygen species generation, which promotes angiogenesis and fibroblast differentiation into myofibroblasts. Furthermore, the levels of growth factors such as FGF‐7, transforming growth factor‐β (TGF‐β), FGF‐2, and IL‐8 are elevated.^23^, ^24^ Kobayashi et al. evaluated prostatic blood flow in aging rats with and without DM (OLETF and LETO rats, respectively). Among rats aged 48 weeks, prostatic blood flow was significantly decreased in OLETF rats compared with LETO rats; blood flow was improved by tadalafil treatment. Furthermore, the protein expression levels of proinflammatory cytokines (e.g., IL‐6, IL‐8, and tumor necrosis factor‐α) were significantly increased in the prostate of OLETF rats; these levels were reduced by tadalafil treatment.^14^ These findings suggest that prostatic ischemia can exacerbate prostatic inflammation; however, cytokine suppression could also be directly caused by tadalafil. Further studies are required to clarify the relationship between ischemia and prostatic inflammation.

## URINE REFLUX

Urine reflux into the prostatic ducts and stroma can also trigger prostatic inflammation. During urination, urine can reportedly reflux into the prostatic glandular ducts, causing chemical irritation and inflammation. However, it is unclear whether prostatic enlargement precedes urine reflux or urine reflux precedes prostatic enlargement. In vitro dye injection into the rat's external urethral orifice showed that the dye could reflux into the prostatic glandular ducts and even into interstitial tissue. Furthermore, urine injection into the external urethral orifice resulted in the proliferation of prostatic interstitial tissue and increased levels of inflammatory cytokines such as IL‐1α, IL‐1β, IL‐6, and tumor necrosis factor‐α.^25^ The urine reflux‐related worsening of inflammation may involve decreased expression of intercellular adhesion substances between prostate glandular epithelial cells. Studies comparing tissues from healthy individuals of various ages and patients with BPH revealed that the expression of claudin‐1, a component of intercellular tight junctions, decreased with age and was significantly lower in patients with BPH.^26^ Furthermore, inflammation itself may be involved in the decreased expression of tight junction proteins. In vitro studies using normal prostate epithelial and BPH cell lines have investigated TGF‐β‐induced changes in the expression levels of E‐cadherin and claudin‐1 between cells, as well as the function of the intercellular epithelial barrier, using trans‐epithelial electrical resistance measurement assays or fluorescein isothiocyanate–dextran Transwell permeability assays. Although E‐cadherin expression was not affected in the TGF‐β‐treated group, the expression of claudin‐1 was suppressed; epithelial barrier function also decreased.^27^

## AUTOIMMUNITY

A possible autoimmune effect on the progression of prostatic inflammation has also been noted. Vickman et al. investigated relationships between autoimmune diseases and BPH; they demonstrated that the prevalence of BPH was significantly higher in patients with autoimmune diseases than in patients without autoimmune diseases.^28^ Thus far, specific autoantigens causing BPH‐related prostatic inflammation have not been identified. Several studies have indicated that prostate‐specific antigen (PSA) and prostatic acid phosphatase (PAP) could be autoimmunity targets.^29^, ^30^ Lokant et al. reported elevated levels of anti‐PSA antibodies in 59% of patients with BPH, but no such elevation was observed in controls. Kouiavskaia showed that PAP173‐192, a PAP‐derived peptide, was more frequently identified in patients with prostatitis than in controls. However, considering the limited research regarding these topics, it is difficult to classify PSA and PAP as autoantibodies. Other substances have been investigated, but the notion that autoantibodies contribute to BPH progression requires further research.

BPH exhibits age‐related progression, implying that multiple factors are involved. Potential mechanisms contributing to increased prostatic inflammation include age‐related decreases in male hormones, pelvic ischemia exacerbation because of arteriosclerosis progression, and increased urine reflux due to the decreased expression of tight junction proteins between prostate glandular epithelial cells. Further investigations are needed to elucidate these processes.

## CHRONIC PROSTATIC INFLAMMATION AND LUTS

BPH is characterized by increases in the numbers of prostatic epithelial and stromal cells, resulting in prostatic enlargement, BOO worsening, and LUTS exacerbation. Three major factors associated with BPH and LUTS are prostatic enlargement, prostatic smooth muscle tension, and prostatic inflammation.^7^ Some studies have demonstrated that prostatic inflammation has a substantial effect on male LUTS severity. In the REDUCE trial, Nickel et al. assessed the degree of prostatic inflammation in hematoxylin and eosin‐stained prostatic tissue and examined its relationship with LUTS severity using the International Prostate Symptom Score (IPSS). Patients with chronic prostatic inflammation had significantly higher total IPSS scores and sub‐scores compared with patients who lacked chronic prostatic inflammation (total IPSS 8.8 vs. 8.2, *p* < 0.0001; irritative subscore 4.3 vs. 4.1, *p* < 0.0001; obstructive subscore 4.4 vs. 4.2, *p* = 0.0013; nocturia subscore 1.6 vs. 1.5, *p* = 0.0005). These results indicated that chronic prostatic inflammation exacerbates LUTS in patients in BPH.^7^

However, the REDUCE trial did not objectively evaluate the association of chronic prostatic inflammation with BPH. Additionally, differences in IPSS scores between groups were very small, even when they were statistically significant. Although this study indicated a possible impact of prostatic inflammation on male LUTS, the actual effects on chronic inflammation within the prostate should be interpreted cautiously based on the results of other studies.

We assessed the relationship between prostatic inflammation and LUTS severity in patients with BPH using the HEV‐like vessel number, determined by immunostaining with MECA‐79, a specific marker for HEVs.^31^ We counted the number of HEV‐like vessels in 86 patients with BPH who underwent surgery; the number of HEV‐like vessels in prostatic tissue was significantly correlated with objective urodynamic parameters related to BOO and bladder storage function.^3^ These results indicated that chronic prostatic inflammation induces voiding dysfunction and storage dysfunction in patients with BPH.

Several studies have shown that the location of lymphocytic inflammation in stromal or nonstromal prostatic tissues is a potentially important factor involved in BOO worsening among patients with BPH. We compared the relationship between clinical parameters of patients with BPH with the location of inflammation and found that the BOO of patients with BPH with stromal prostatic inflammation was more severe than that in those with nonstromal prostatic inflammation, whereas prostate volume was not the factor exacerbating BOO in multivariate analysis.^32^

Taken together, our findings suggest that chronic prostatic inflammation of the stroma is one of a key factor in exacerbation among multiple LUTDs; however, further studies are required to fully clarify the impact of prostatic inflammation on LUTS.

## POTENTIAL THERAPEUTIC APPROACHES FOR PROSTATIC INFLAMMATION

### Assessment of prostatic inflammation

As described above, the management of prostatic inflammation may be an effective therapeutic approach for BPH because prostatic inflammation contributes to the exacerbation of both voiding and storage symptoms. To treat prostatic inflammation, the degree of inflammation must be assessed; various assessment methods have been reported, including histological examination of prostatic tissue^3^, ^7^ and measurement of serum PSA.^33^, ^34^ However, considering challenges regarding measurement, cost, specificity, and invasiveness, broad use of these methods in clinical settings is impractical.

**FIGURE 2:**
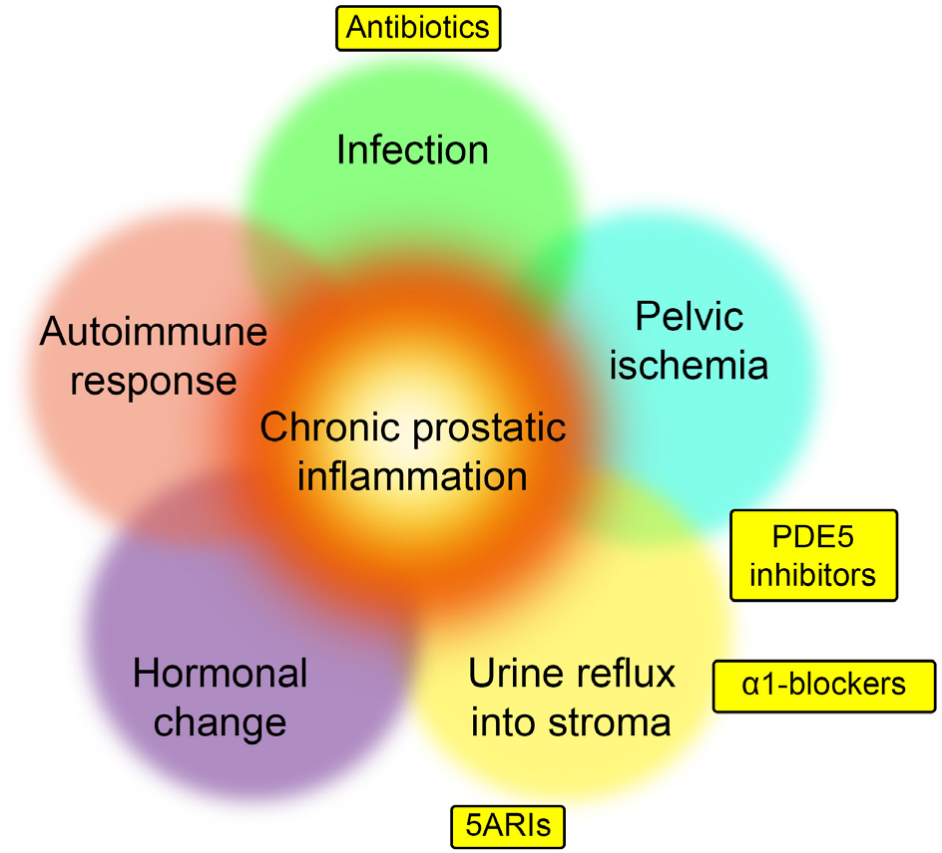
The relationships between causes of prostatic inflammation and medications for BPH. This figure depicts current treatments for BPH that address prostatic inflammation, in relation to their therapeutic mechanisms and various potential etiologies of prostatic inflammation.

Infection, pelvic ischemia, urine reflux into the stroma, hormonal changes, and autoimmune responses are known mechanisms underlying chronic prostatic inflammation. Various treatments can be considered based on the pathophysiology of prostatic chronic inflammation; pharmacological therapies include antibiotics, α‐blockers, phosphodiesterase 5 inhibitors, 5ARIs, and pollen extracts. However, there is no standard evidence‐based treatment. Additionally, these pathophysiological conditions overlap with each other, and multimodal therapies for chronic prostatic inflammation are needed (Figure 2). Possible treatments for BPH‐related prostatic inflammation are very similar to treatments for CP/CPPS because their pathophysiologies may be identical in terms of prostatic inflammation. Although the treatments we present below are common for BPH and CP/CPPS, they are considered investigational with respect to prostatic inflammation and should be used cautiously in clinical practice.

### Antibiotics

Bacterial involvement is a potential etiology of prostatic inflammation. Such patients are diagnosed with chronic bacterial prostatitis, defined as category II CP/CPPS. In cases of bacterial infection, treatment with antimicrobial agents can be effective; typically, oral antimicrobial agents (e.g., fluoroquinolones) are administered for more than 6 weeks.^35^ However, in most patients with chronic prostatitis, urinalyses do not show pyuria or bacteriuria, even after prostate massage. Nonetheless, if the symptoms are relieved by treatment with antibiotics, the patient is typically diagnosed with chronic bacterial prostatitis. Nickel et al. evaluated the effects of treatment with antibiotics in patients with category II or III chronic prostatitis; they found that National Institute of Health Chronic Prostatitis Symptom Index (NIH–CPSI) scores improved after 12 weeks of ofloxacin administration. A patient with category III prostatitis may benefit from antibiotics through the eradication of microorganisms that cannot be cultured by conventional methods, along with the cytokine suppression induced by antibiotics.^36^ Thus, it is reasonable for patients with prostatic inflammation to receive treatment with antibiotics.

### α1‐blockers

Conventional treatments for BPH include α1‐blockers, which induce smooth muscle relaxation in the prostate, bladder neck, and urethra, thereby relieving BOO. Although α1‐blockers do not directly suppress prostatic inflammation, experiments in rats have shown that silodosin treatment reduces urine reflux into prostatic ducts and stroma, thus reducing prostatic inflammation.^25^ These medications may also suppress prostatic inflammation by relieving prostatic smooth muscle tension and improving blood flow.

### Phosphodiesterase 5 inhibitors

Phosphodiesterase 5 inhibitors represent another therapeutic option for BPH with various effects, including the suppression of prostatic inflammation. Our previous study using the OLETF rat model of DM revealed that tadalafil suppressed prostatic inflammation by improving blood flow.^14^ Moreover, tadalafil reduced the levels of 8‐OHdG, an oxidative stress marker. Finally, tadalafil‐mediated activation of the nitric oxide/cyclic guanosine monophosphate signaling pathway might also have an anti‐inflammatory effect in prostatic tissues. Thus, tadalafil is a leading candidate for use in the treatment of prostatic inflammation.

### 5ARIs

5ARIs represent another treatment option for patients with BPH. During 5ARI treatment, prostate volume is reduced by inhibiting the conversion of testosterone to dihydrotestosterone in prostatic tissues. Dutasteride treatment reportedly improved symptoms in patients with CP/CPPS^37^; however, the anti‐inflammatory effects of 5ARIs were not pathologically evaluated in that study. Another study showed that androgen receptor activation suppressed inflammation in BPH.^38^ Further research is needed to clarify the associations of androgens with prostatic inflammation.

### Pollen extract

Several plant extract‐based drugs have been used for BPH and CP/CPPS. Some drugs are not supported by scientific evidence, whereas others have demonstrated clinical efficacy. Cernitin pollen extract (cernilton) is widely used to manage BPH and CP/CPPS. Anti‐inflammatory effects and delayed BPH progression are the main reported effects of cernilton.^39^ Among patients with CP/CPPS resistant to α1 blockers, cernilton was more effective than tadalafil in reducing pain scores, whereas tadalafil was more effective in improving LUTS.^40^ Furthermore, patients with CP/CPPS receiving pollen extract and vitamins had better symptom scores and seminal plasma levels of IL‐6, IL‐8, and IL‐10 compared with such patients receiving bromelain.^41^ Thus, pollen extract may be an effective treatment for prostatic inflammation.

### Nonsteroidal anti‐inflammatory drugs (NSAIDs) and steroids

In terms of suppressing inflammation, a physician might initially consider NSAIDs and steroids; however, there have been no reports in which these medications alleviated chronic prostatitis symptoms or inflammation. A comparison of NSAIDs and placebo in patients with CP/CPPS revealed no significant difference in symptom scores.^42^ Similarly, a comparison between prednisolone and placebo showed no significant symptom improvement.^43^

## CONCLUSIONS

Chronic prostatic inflammation can worsen urinary and storage symptoms, potentially decreasing the quality of life in patients with BPH. Efforts to understand the mechanisms underlying chronic inflammation should be a key component of BPH research; further advancements in this area are expected regarding pathophysiological mechanisms and novel treatment methods.

## CONFLICT OF INTEREST STATEMENT

All authors have no COI regarding this article.

## AUTHOR CONTRIBUTIONS

**So Inamura:** Data curation; formal analysis; investigation; methodology; resources; software; writing – original draft. **Naoki Terada:** Conceptualization; funding acquisition; project administration; supervision; validation; visualization; writing – review and editing.

## References

[1] Sakai Y , Kobayashi M . Lymphocyte ‘homing’ and chronic inflammation. Pathol Int. 2015;65:344–354. 25831975 10.1111/pin.12294

[2] Furman D , Campisi J , Verdin E , Carrera‐Bastos P , Targ S , Franceschi C , et al. Chronic inflammation in the etiology of disease across the life span. Nat Med. 2019;25:1822–1832. 31806905 10.1038/s41591-019-0675-0 PMC7147972

[3] Inamura S , Shinagawa T , Hoshino H , Sakai Y , Imamura Y , Yokoyama O , et al. Appearance of high endothelial venule‐like vessels in benign prostatic hyperplasia is associated with lower urinary tract symptoms. Prostate. 2017;77:794–802. 28181681 10.1002/pros.23319

[4] Chen L , Zhang M , Liang C . Chronic prostatitis and pelvic pain syndrome: another autoimmune disease? Arch Immunol Ther Exp. 2021;69:24. 10.1007/s00005-021-00628-3 34523016

[5] Nickel JC , Elhilali M , Vallancien G , Group A‐OS . Benign prostatic hyperplasia (BPH) and prostatitis: prevalence of painful ejaculation in men with clinical BPH. BJU Int. 2005;95:571–574. 15705082 10.1111/j.1464-410X.2005.05341.x

[6] Marker PC , Donjacour AA , Dahiya R , Cunha GR . Hormonal, cellular, and molecular control of prostatic development. Dev Biol. 2003;253:165–174. 12645922 10.1016/s0012-1606(02)00031-3

[7] Nickel JC , Roehrborn CG , O'Leary MP , Bostwick DG , Somerville MC , Rittmaster RS . The relationship between prostate inflammation and lower urinary tract symptoms: examination of baseline data from the REDUCE trial. Eur Urol. 2008;54:1379–1384. 18036719 10.1016/j.eururo.2007.11.026 PMC2643127

[8] Andriole G , Bostwick D , Brawley O , Gomella L , Marberger M , Tindall D , et al. Chemoprevention of prostate cancer in men at high risk: rationale and design of the reduction by dutasteride of prostate cancer events (REDUCE) trial. J Urol. 2004;172:1314–1317. 15371831 10.1097/01.ju.0000139320.78673.2a

[9] Li J , Li Y , Cao D , Huang Y , Peng L , Meng C , et al. The association between histological prostatitis and benign prostatic hyperplasia: a single‐center retrospective study. Aging Male. 2022;25:88–93. 35289705 10.1080/13685538.2022.2050360

[10] Kobayashi M , Mitoma J , Nakamura N , Katsuyama T , Nakayama J , Fukuda M . Induction‐of‐peripheral‐lymph‐node‐addressin‐in‐human‐gastric‐mucosa‐infected‐by‐helicobacter. Proc Natl Acad Sci USA. 2004;101:17807–17812. 15591109 10.1073/pnas.0407503101 PMC539746

[11] Kobayashi M , Hoshino H , Masumoto J , Fukushima M , Suzawa K , Kageyama S , et al. GlcNAc6ST‐1‐mediated decoration of MAdCAM‐1 protein with L‐selectin ligand carbohydrates directs disease activity of ulcerative colitis. Inflamm Bowel Dis. 2009;15:697–706. 19067429 10.1002/ibd.20827 PMC2696616

[12] De Nunzio C , Kramer G , Marberger M , Montironi R , Nelson W , Schröder F , et al. The controversial relationship between benign prostatic hyperplasia and prostate cancer: the role of inflammation. Eur Urol. 2011;60:106–117. 21497433 10.1016/j.eururo.2011.03.055

[13] Penna G , Fibbi B , Amuchastegui S , Cossetti C , Aquilano F , Laverny G , et al. Human benign prostatic hyperplasia stromal cells as inducers and targets of chronic immuno‐mediated inflammation. J Immunol. 2009;182:4056–4064. 19299703 10.4049/jimmunol.0801875

[14] Kobayashi H , Zha X , Nagase K , Inamura S , Taga M , Aoki Y , et al. Phosphodiesterase 5 inhibitor suppresses prostate weight increase in type 2 diabetic rats. Life Sci. 2022;298:120504. 35367242 10.1016/j.lfs.2022.120504

[15] Nickel JC , Roehrborn CG , Castro‐Santamaria R , Freedland SJ , Moreira DM . Chronic prostate inflammation is associated with severity and progression of benign prostatic hyperplasia, lower urinary tract symptoms and risk of acute urinary retention. J Urol. 2016;196:1493–1498. 27378134 10.1016/j.juro.2016.06.090

[16] Akiho H , Tokita Y , Nakamura K , Satoh K , Nishiyama M , Tsuchiya N , et al. Involvement of interleukin‐17A‐induced hypercontractility of intestinal smooth muscle cells in persistent gut motor dysfunction. PLoS One. 2014;9:e92960. 24796324 10.1371/journal.pone.0092960 PMC4010403

[17] Govindaraju V , Michoud MC , Ferraro P , Arkinson J , Safka K , Valderrama‐Carvajal H , et al. The effects of interleukin‐8 on airway smooth muscle contraction in cystic fibrosis. Respir Res. 2008;9:76. 19046427 10.1186/1465-9921-9-76 PMC2633308

[18] Yokoyama O , Yusup A , Oyama N , Aoki Y , Miwa Y , Akino H . Improvement in bladder storage function by tamsulosin depends on suppression of C‐fiber urethral afferent activity in rats. J Urol. 2007;177:771–775. 17222679 10.1016/j.juro.2006.09.076

[19] Funahashi Y , Takahashi R , Mizoguchi S , Suzuki T , Takaoka E , Ni J , et al. Bladder overactivity and afferent hyperexcitability induced by prostate‐to‐bladder cross‐sensitization in rats with prostatic inflammation. J Physiol. 2019;597:2063–2078. 30666643 10.1113/JP277452 PMC6441933

[20] Bajic P , Dornbier RA , Doshi CP , Wolfe AJ , Farooq AV , Bresler L . Implications of the genitourinary microbiota in prostatic disease. Curr Urol Rep. 2019;20:34. 31104156 10.1007/s11934-019-0904-6

[21] Takezawa K , Fujita K , Matsushita M , Motooka D , Hatano K , Banno E , et al. The firmicutes/Bacteroidetes ratio of the human gut microbiota is associated with prostate enlargement. Prostate. 2021;81:1287–1293. 34516694 10.1002/pros.24223

[22] Mukaida N , Okamoto S , Ishikawa Y , Matsushima K . Molecular mechanism of interleukin‐8 gene expression. J Leukoc Biol. 1994;56:554–558. 7525815

[23] Kozlowski R , Kershen R , Siroky M , Krane R , Azadzoi K . Chronic ischemia alters prostate structure and reactivity in rabbits. J Urol. 2001;165:1019–1026. 11176533

[24] Wang L , Yang JR , Yang LY , Liu ZT . Chronic inflammation in benign prostatic hyperplasia: implications for therapy. Med Hypotheses. 2008;70:1021–1023. 17935901 10.1016/j.mehy.2007.08.022

[25] Funahashi Y , Majima T , Matsukawa Y , Yamamoto T , Yoshida M , Gotoh M . Intraprostatic reflux of urine induces inflammation in a rat. Prostate. 2017;77:164–172. 27683251 10.1002/pros.23257

[26] Pascal LE , Dhir R , Balasubramani GK , Chen W , Hudson CN , Srivastava P , et al. Claudin‐1 down‐regulation in the prostate is associated with aging and increased infiltration of inflammatory cells in BPH. Am J Clin Exp Urol. 2021;9:53–64. 33816694 PMC8012836

[27] Feng Li LEP , Wang K , Zhou Y , Balasubramani GK , O'Malley KJ , Dhir R , et al. Transforming growth factor Beta 1 impairs benign prostatic luminal epithelial cell monolayer barrier function. Am J Clin Exp Urol. 2020;25:9–17. PMC7076294 32211449

[28] Vickman RE , Aaron‐Brooks L , Zhang R , Lanman NA , Lapin B , Gil V , et al. TNF is a potential therapeutic target to suppress prostatic inflammation and hyperplasia in autoimmune disease. Nat Commun. 2022;13:2133. 35440548 10.1038/s41467-022-29719-1 PMC9018703

[29] Lokant MT , Naz RK . Presence of PSA auto‐antibodies in men with prostate abnormalities (prostate cancer/benign prostatic hyperplasia/prostatitis). Andrologia. 2015;47:328–332. 24620795 10.1111/and.12265

[30] Kouiavskaia DV , Southwood S , Berard CA , Klyushnenkova EN , Alexander RB . T‐cell recognition of prostatic peptides in men with chronic prostatitis/chronic pelvic pain syndrome. J Urol. 2009;182:2483–2489. 19765754 10.1016/j.juro.2009.07.067 PMC2760636

[31] Yeh JC , Hiraoka N , Petryniak B , Nakayama J , Ellies LG , Rabuka D , et al. Novel sulfated lymphocyte homing receptors and their control by a Core1 extension beta 1,3‐N‐acetylglucosaminyltransferase. Cell. 2001;105:957–969. 11439191 10.1016/s0092-8674(01)00394-4

[32] Nickel JC , True LD , Krieger JN , Berger RE , Boag AH , Young ID . Consensus development of a histopathological classificationsystem for chronic prostatic inflammation. BJU Int. 2001;87:797–805. 11412216 10.1046/j.1464-410x.2001.02193.x

[33] Irani J , Levillain P , Goujon JM , Bon D , Dore B , Aubert J . Inflammation in benign prostatic hyperplasia: correlation with prostate specific antigen value. J Urol. 1997;157:1301–1303. 9120926 10.1016/s0022-5347(01)64957-7

[34] Liao X , Tang Z , Ai J , Xu H , Zhang S , Liu L , et al. Detection of prostatic inflammation from peripheral lymphocyte count and free/Total PSA ratio in men with LUTS/BPH. Front Pharmacol. 2020;11:589. 32425801 10.3389/fphar.2020.00589 PMC7204507

[35] Appiya Santharam M , Khan FU , Naveed M , Ali U , Ahsan MZ , Khongorzul P , et al. Interventions to chronic prostatitis/chronic pelvic pain syndrome treatment. Where are we standing and what's next? Eur J Pharmacol. 2019;857:172429. 31170381 10.1016/j.ejphar.2019.172429

[36] Nickel JC , Downey J , Johnston B , Clark J , Group CPR . Predictors of patient response to antibiotic therapy for the chronic prostatitis/chronic pelvic pain syndrome: a prospective multicenter clinical trial. J Urol. 2001;165:1539–1544. 11342913

[37] Nickel JC , Roehrborn C , Montorsi F , Wilson TH , Rittmaster RS . Dutasteride reduces prostatitis symptoms compared with placebo in men enrolled in the REDUCE study. J Urol. 2011;186:1313–1318. 21849186 10.1016/j.juro.2011.05.071

[38] Vignozzi L , Cellai I , Santi R , Lombardelli L , Morelli A , Comeglio P , et al. Antiinflammatory effect of androgen receptor activation in human benign prostatic hyperplasia cells. J Endocrinol. 2012;214:31–43. 22562653 10.1530/JOE-12-0142

[39] Kamijo T , Sato S , Kitamura T . Effect of cernitin pollen‐extract on experimental nonbacterial prostatitis in rats. Prostate. 2001;49:122–131. 11582591 10.1002/pros.1126

[40] Matsukawa Y , Naito Y , Funahashi Y , Ishida S , Fujita T , Tochigi K , et al. Comparison of cernitin pollen extract vs tadalafil therapy for refractory chronic prostatitis/chronic pelvic pain syndrome: a randomized, prospective study. Neurourol Urodyn. 2020;39:1994–2002. 32648985 10.1002/nau.24454

[41] Cai T , Verze P , La Rocca R , Palmieri A , Tiscione D , Luciani LG , et al. The clinical efficacy of pollen extract and vitamins on chronic prostatitis/chronic pelvic pain syndrome is linked to a decrease in the pro‐inflammatory cytokine interleukin‐8. World J Mens Health. 2017;35:120–128. 28497911 10.5534/wjmh.2017.35.2.120 PMC5583369

[42] Nickel JC , Shoskes DA , Wagenlehner FM . Management of chronic prostatitis/chronic pelvic pain syndrome (CP/CPPS): the studies, the evidence, and the impact. World J Urol. 2013;31:747–753. 23568442 10.1007/s00345-013-1062-y

[43] Bates SM , Hill VA , Anderson JB , Chapple CR , Spence R , Ryan C , et al. A prospective, randomized, double‐blind trial to evaluate the role of a short reducing course of oral corticosteroid therapy in the treatment of chronic prostatitis/chronic pelvic pain syndrome. BJU Int. 2007;99:355–359. 17313424 10.1111/j.1464-410X.2007.06667.x

